# The effects of flipped classrooms on undergraduate pharmaceutical marketing learning: A clustered randomized controlled study

**DOI:** 10.1371/journal.pone.0214624

**Published:** 2019-04-10

**Authors:** Yuan He, Jun Lu, Huaxing Huang, Shutong He, Nina Ma, Zimo Sha, Yanjun Sun, Xin Li

**Affiliations:** 1 Department of Psychology, School of Humanities and Social Sciences, Nanjing Medical University, Nanjing, Jiangsu, the People's Republic of China; 2 Department of Surgery, The First School of Clinical Medicine,Nanjing Medical University, Nanjing, Jiangsu, the People's Republic of China; 3 Office of Academic Affairs, Kangda College, Nanjing Medical University, Lianyungang, Jiangsu, the People's Republic of China; 4 Department of Psychiatry, The Fourth School of Clinical Medicine, Nanjing Medical University, Nanjing, Jiangsu, the People's Republic of China; 5 Department of Clinical Pharmacy, School of Pharmacy, Nanjing Medical University, Nanjing, Jiangsu, the People's Republic of China; 6 Department of Health Policy, School of Health Policy and Management, Nanjing Medical University, Nanjing, Jiangsu, the People's Republic of China; 7 Center for Global Health, School of Public Health, Nanjing Medical University, Nanjing, Jiangsu, the People's Republic of China; University of Westminster, UNITED KINGDOM

## Abstract

**Background:**

Recently, flipped classrooms (FCs) have gradually been used in Chinese higher education settings. However, few studies have focused on the effects of FCs on interdisciplinary curricula. The purpose of this study was to examine the impact of an FC on the engagement, performance, and perceptions of students and on teacher-student interaction in a pharmaceutical marketing course.

**Design:**

A clustered randomized controlled study was conducted, with 137 junior-year pharmacy undergraduates using an FC serving as the intervention group, in contrast to students using lecture-based learning (LBL) as the control group. Flanders’ interaction analysis system (FIAS) was used to measure teacher-student interaction, and questionnaires regarding attitudes toward and satisfaction with the teaching model were administered.

**Results:**

The students in the FC group scored significantly higher than those in the LBL group (88.21±5.95 vs. 80.05±5.59, t = -8.08, *p* = 0.000) on pharmaceutical marketing. The multiple linear regression results showed that the FC model had a significant impact on student performance (β = 8.16, *p*<0.0001). The percentages of teacher talk in the FC and LBL groups were 21% and 96%, respectively (χ^2^ = 2170.274, *p* = 0.000); however, the percentages of student talk in the FC and LBL groups were 75% and 2.6%, respectively (χ^2^ = 2012.483, *p* = 0.000). Compared with the LBL group, most students in the FC group held more positive attitudes toward the teaching model; the mean scores for the 8 attitude attributes in the FC group were significantly higher than those in the LBL group (*p* = 0.000). There were significant differences in the ratings of satisfaction with teacher-student interaction (*p* = 0.000), the students’ learning attitude (*p* = 0.000), the teacher’s preparatory work (*p* = 0.000), the teaching objective (*p* = 0.000), and the teaching effect (*p* = 0.000) between the two groups.

**Conclusion:**

Compared with LBL methods, implementing the FC model improved student performance, increased teacher-student interaction and generated positive student attitudes toward the experience. As an effective pedagogical model, it can also stimulate pharmacy students’ learning interest and improve their self-learning abilities.

## Introduction

Concurrent with the increasing use of information technology and electronic learning (e-learning) approaches, flipped classrooms (FCs) are growing in popularity in higher education worldwide. Previous scoping reviews, which summarized the relevant research regarding the emergence of the FC approach, showed that much convincing evidence of improved course grades and learning satisfaction with the active learning or FC approach was emerging [1–8]. However, most of the studies included in the reviews were conducted in Western countries such as the United States and Australia. Only a few studies in East Asian countries have evaluated the impacts of the FC model on student satisfaction [1–8]. To date, studies that focused on FC teaching methods primarily came from Western contexts. The enormous cultural differences between Western and Asian populations could give rise to potential cultural conflicts and exert negative effects on the FC teaching model. It is necessary to expand the understanding of how the FC teaching model works in other cultural contexts, especially in China, the largest developing country worldwide.

According to statistics from China’s Department of Education, approximately 40% of Chinese pharmacy undergraduates have a willingness to engage in pharmaceutical marketing and management, becoming, for example, sales and marketing managers, medical representatives or medical product managers [9]. In China, pharmaceutical marketing is listed as one of the compulsory courses in the curriculum of pharmacy education [10]. The specialized pharmaceutical marketing course focuses exclusively on the pharmaceutical industry or health-related field, and it not only offers the same analytical and financial skills as a general marketing course but also emphasizes the knowledge and skills needed to understand the economic, financial and organizational structure unique to the pharmaceutical industry [11].

Specifically, the aims of this course are to teach pharmacy students to master the principles of marketing, economics, management theory and evaluation of marketing cases in the pharmaceutical industry. At the end of the curriculum, pharmacy students can understand and have mastery of the knowledge and skills associated with pharmaceutical marketing planning, implementation and surveying.

Traditionally, the lecture-based learning (LBL) method has been widely used to teach the undergraduate pharmacy curriculum in Chinese university classrooms. However, in classic LBL sessions, the teacher is regarded as the highly knowledgeable leader who can control the process of teaching in the classroom, while students are usually viewed as passive learners who only receive knowledge from the teacher [12,13]. As LBL is used as the only teaching method, students in lecture courses are passive recipients. This passivity leads to negative effects in which students usually perceive a lack of opportunity to take more initiative in analyzing problems and developing problem-solving skills [14, 15]. Furthermore, it almost always takes the teacher a great deal of time and effort to communicate theoretical knowledge and basic contents to the students in the classroom. On the one hand, teachers cannot satisfy the personal needs of each learner. On the other hand, students do not have enough time to receive one-on-one guidance or to obtain problem-based learning experience [16, 17]. In particular, under the LBL model, teachers usually pay little to no attention to which practical abilities students can improve. Furthermore, this passive learning process inevitably leads to student boredom and prevents students from acquiring the necessary application and communication skills [18–19].

Therefore, to transform students into “active self-explorers of knowledge” and to promote their active participation in the learning process, it is very important to develop or seek new teaching models that have the ability to improve the teaching effect during a limited period of time [4,13,18,19].

The FC is a new pedagogical method that complements traditional teaching models; it employs asynchronous video lectures and practice problems as homework and active, group-based problem-solving activities in the classroom [20,21]. In an FC, the teacher is no longer the “speaker” on the podium; instead, the teacher becomes a coordinator for the students. Some scholars have described the nature of the flipped teaching mode as follows: By blending the strengths of internet-driven instruction outside the classroom (e.g., digital videos, self-regulated learning, online discussions) and face-to-face inside the classroom (e.g., collaborative study, applied problem-solving, instructor and peer engagement), the FC model can effectively increase student engagement, improve student performance and strengthen the development of creative thinking [22–25]. For instance, as early as 1996, an education study conducted in the United States that changed the before, during and after class traditional teaching order demonstrated that the “inverted classroom” was able to effectively engage students in the learning process [20]. In the FC context, students can not only selectively watch online videos based on their knowledge levels but also freely replay core and difficult content. In the classroom, teachers and students have sufficient time to conduct face-to-face discussions. Meanwhile, to enhance knowledge integration and application, students are asked to use in-class time for group discussion. These discussions, especially those involving controversial topics, can play an important role in improving students’ understanding of the key points of the course. [26]. Under the flipped pedagogy model, students can benefit from the freedom to choose the most appropriate method to learn knowledge before class. Most importantly, this model integrates the process of knowledge internalization into class time to enhance the extensive learning cooperation between students and to increase teacher-student interaction. The “negative recipients” in traditional classroom learning can be transformed into “positive explorers” under flipped teaching through a recognition of the individual characteristics of cognitive learning [26].

Research on the use of FCs in the field of pharmacy education has grown over the past few years. [27–31]. McLaughlin *et al*. found that the flipped pharmacy classroom enhanced the quality of students’ experiences in a basic pharmaceutics course through a thoughtful course design, enriched dialogue, and the promotion of learner autonomy [27]. They also demonstrated that engagement with a highly interactive online preparatory tool was positively related to student learning in neurologic pharmacotherapy [28]. Pierce and Fox also revealed that the positive outcomes of an FC model for teaching a renal pharmacotherapy module resulted in improved student performance and favorable student perceptions of the instructional approach [30]. However, a study by Bossaer *et al*. involving a pharmacotherapy oncology module found that examination scores were poorer in the FC cohort than in the traditional LBL cohort [31].

Recently, teachers have been encouraged to initiate teaching innovation programs using FCs in Chinese higher educational contexts [32]. For instance, Zeng *et al*. implemented a randomized controlled study on undergraduate electrocardiogram learning. Compared with those learning in the LBL classroom, the medical students learning through the FC model reported greater self-learning abilities, increased interest and decreased boredom [33]. However, few studies have highlighted the effect of the FC model on interdisciplinary curricula, such as pharmaceutical marketing.

In this study, we define the FC as an educational technique that consists of two parts: direct web-based individual self-learning outside the classroom and interactive group learning activities inside the classroom. Specifically, the primary objective of the current study is to use mixed methods to examine the impact of FC teaching on the engagement, performance, and perceptions of junior-year pharmacy students and on teacher-student interaction with a flipped pharmaceutical marketing course. The secondary objectives include serving as a pilot for pedagogy researchers to implement and develop the FC methodology for pharmaceutical marketing and providing a theoretical foundation that will inform future innovative course design and a broader range of FC applications in the pharmacy curriculum.

## Methods

### Study design and participants

We conducted a clustered randomized controlled study on student performance, student satisfaction and teacher-student interaction, with the class as the unit of analysis. This study was undertaken at Nanjing Medical University (NMU) in China between September and December 2017.

The participants were recruited from the School of Pharmacy at NMU in August 2017. The inclusion criteria were (1) majoring in pharmacy, (2) being aged ≥18 years, and (3) being willing to sign a consent form to participate in the study. Students were excluded if they had transferred from other specialties or schools, were transferring to other specialties or schools for further study, or had already taken the pharmaceutical marketing course prior to this study. During the introductory session, the researcher assistant (RA), who was not involved in student outcomes or examinations, followed a standardized script to give the full details of the study to the students and asked for informed consent. The RA emphasized that participation was completely voluntary and would be independent of the students’ course grades. After distributing the informed consent forms to the students, the RA asked them to read the form and organized a discussion session regarding the study. After the introductory session, the students had at least 24 hours to decide whether to participate in the study. Moreover, they themselves submitted the informed consent form in a sealed file packet to the RA at the end of the recruitment period.

This study determined the sample size according to the rule of statistical power [34,35,18,19]. The sample size was determined using G*Power 3.1 for Windows. For the purposes of this study, the significance level was set at 0.05 (two-sided) with 80% statistical power and the effect size at 0.5 for analysis. Considering a 10% dropout rate, a minimum number of 53 pairs (106 participants) would be sufficient to evaluate the outcome. In this study, randomization was performed at the class level. Specifically, this study was further undertaken in five independent teaching classes, which were randomized into the control and the FC group. Randomization at the student level and blinding were not possible due to the nature of the teaching model. Three classes were randomly allocated to the FC group, while the other two were assigned to the LBL group. A total of 137 junior-year pharmacy students with five independent teaching classes were enrolled in this study. Eighty-one students participated in the FC group, while fifty-six students participated in the LBL group. This sample size fulfilled the requirement.

### Teaching contents and methods

All participants in the two groups had the same teacher, the same teaching syllabus, and the same textbook. The teacher also set up the same teaching goals, the same teaching schedule and the same test model. This course curriculum covered ten teaching chapters belonging to the pharmaceutical marketing knowledge domain. These chapters addressed the following contents: the definition of pharmaceutical marketing; the concept of marketing; the pharmaceutical marketing mix (the 4Ps); the market environment; consumer behavior; market segmentation, targeting and positioning (STP strategy); communications (personal sales, sales promotion, public relations, publicity, advertising); planning; the product life cycle; and customer focus. In the chapter named “market segmentation, targeting and positioning (STP strategy)”, each group was taught using FC teaching or LBL. Over four consecutive weeks, the classes that were held for the chapter included four teaching units. The details of the teaching methods are as follows:

### FC teaching method

**Fig 1:**
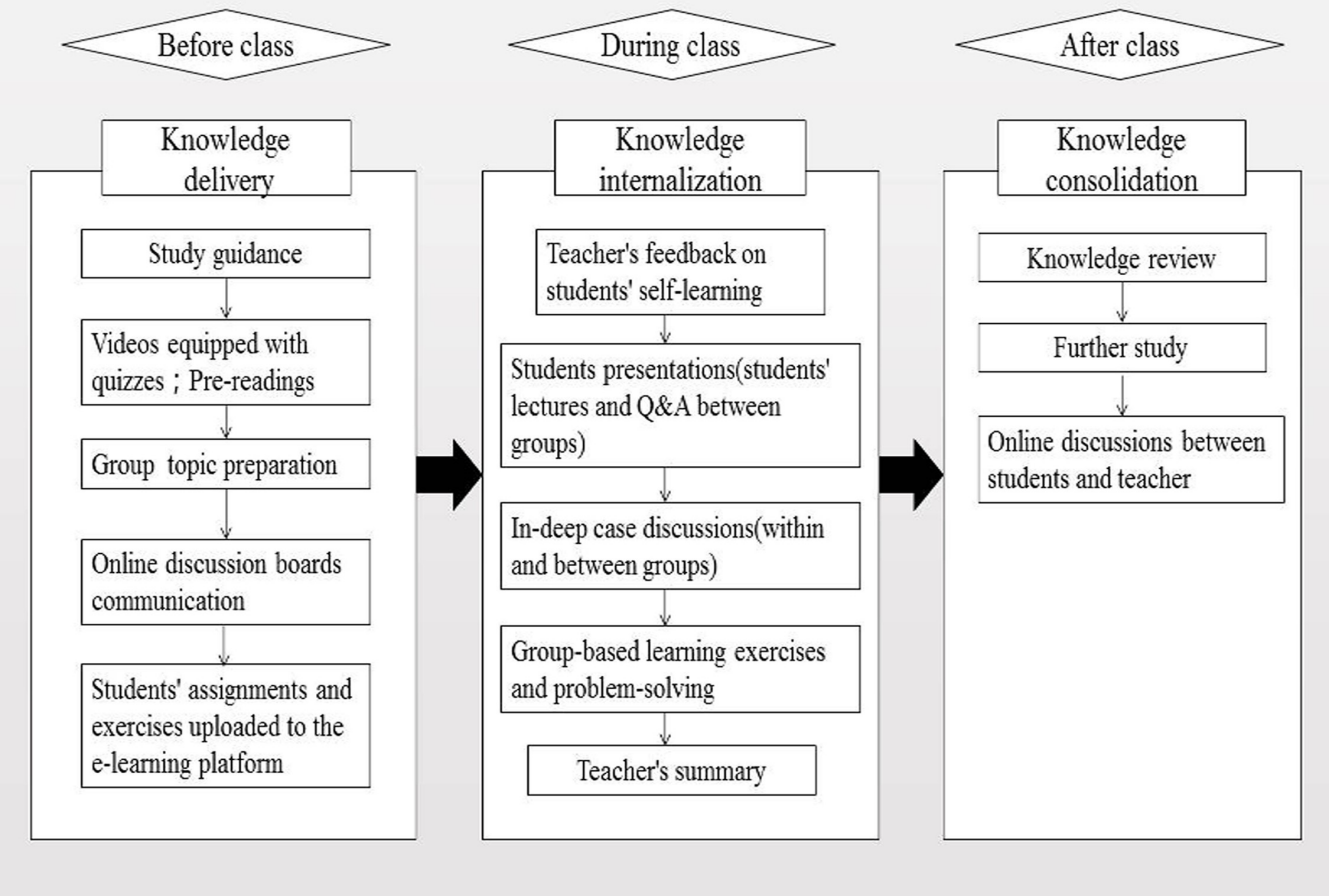
The flipped classroom structure and setting flowchart.

The whole process of FC teaching can be divided into three phases: the knowledge delivery, knowledge internalization and knowledge consolidation phases[35–37]. The process consists of the following (Fig 1):

#### 1. The knowledge delivery phase (out-of-class activities)

In this phase, to successfully carry out knowledge delivery before class, some out-of-class activities should be performed by the teacher and students, including preparation by the teacher and students. Three months prior to the course, the teacher received training for microlecture sessions. Based on the teaching goals and contents, the teacher decomposed his or her knowledge, compiled the PowerPoint courseware, designed and produced the microvideos, and uploaded the videos to the e-learning platform. Each video was accompanied by quizzes and exercises to help students learn by themselves and to test the effect of self-learning.

Three weeks before the classroom was flipped, the teacher introduced the aims of the teaching and the details of curriculum to the students and announced the requirements and procedures of FC learning. To facilitate group discussions, the students were divided into 8 groups. Each group was required to nominate a chair, who was the main point of contact and had overall responsibility for assembling the group, leading discussion within the group, and ensuring the timely completion of work.The chair also coordinated microlesson learning activities and communications to address problems among group members. The students were required to watch narrated microvideos. At the same time, each group was required to prepare a presentation before class time, namely, a talk in which a piece of group work was shown and explained to the teacher and students in the FC. Two weeks prior to each teaching unit, the teacher listed 5–6 presentation topics on the e-learning platform. Each group could select any one of the listed topics. In the FC, each group presented its learning results to the entire class. The students’ presentation grades accounted for 30% of their entire final course grade. Moreover, the teacher uploaded supplementary reading materials, including PowerPoint courseware, textbook sections, self-learning materials and exercise information, and sent the links via the e-learning platform and discussion forums.

The reading materials included a variety of classic marketing books, papers, journals and slides. The topics of the reading materials consisted of broad marketing research, market targeting and market segmentation, the determination of distribution, pricing and promotion strategies, the development of a communications strategy, budgeting, and how to envision long-term market development goals. For instance, the book “*Marketing 4*.*0*: *Moving from Traditional to Digital”*, published by Wiley, was recommended to the students [38]. During the knowledge delivery phase, group members could freely discuss the content of a small private online course (SPOC) and communicate with the members of their own groups anonymously on the website discussion boards. On the day prior to the class for each teaching unit, the chair of each group uploaded group members’ assignments and exercises to the e-learning platform, and subsequently, the teacher could evaluate the effectiveness of self-learning.

#### 2. The knowledge internalization phase (in-class activities)

The in-class activities consisted of three stages. In the first stage, the teacher gave a concise lecture that highlighted the key points of the teaching unit based on the assignments and student feedback. The aim of this stage was to resolve the major problems and confusion shown during students’ self-learning [36]. The first stage lasted approximately 10 minutes.

In the second stage, the role of the teacher was switched from that of a leader to that of a coordinator, and in-class learning started with the student-initiated interactive learning model between the teacher and students [39,40,7,4]. The in-class activities could be carried out with different learning strategies, such as student presentations, group discussions and collaborations, and problem-solving [2,7].

First, each group made a presentation on the topic that the members had chosen. During the presentation process, the students in the other groups were encouraged by the teacher to interrupt the speaker or presenting group and to ask questions. Accordingly, the speaker or the presenting group members gave a preliminary answer to the other groups [41–42,19]. All students could also propose new issues for further discussion between groups. Each group took 8 minutes to complete this task. Second, the teacher offered a teacher-student interaction session in which the students freely raised any concerns regarding the presentations and the teacher briefly asked each group questions on key points. This session also provided an in-depth discussion about the questions that were raised by other groups during the previous presentations. Student concerns could be addressed either by students from other groups or by the teacher. After the group-based discussion, each group chose a representative to answer the questions raised, and the other group members could provide new thoughts about the answers, ask further questions, or discuss between groups [43,7,35]. This session lasted approximately 3–4 minutes for each group. In the second stage, the eight groups took a total of approximately 90 minutes to present ideas and discuss issues.

Finally, in the third stage, the teacher provided simple reviews and summaries; then, the teacher provided some exercises to all the students. Each group solved the problems through discussion within the group [44]. After the teacher presented the key analysis, the representative of each group contributed to the interpretation and supplementary conclusions. The third stage lasted approximately 20 minutes.

#### 3. The knowledge consolidation phase

After the classroom was flipped, the students were able to use the e-learning platform to replay the microvideo lectures. According to their individual level of knowledge of the content, they could also review the key points and marketing cases through online group discussions. Additionally, during online discussions, students could discuss difficult points with the teacher through the e-learning platform.

### Lecture-based learning method

In the control group, traditional classes, known as the LBL method, were used as follows. Two weeks prior to class, the teacher distributed supplementary learning materials, including pharmaceutical marketing teaching materials, classic cases, quizzes and exercises. In the LBL class, the teacher gave a lecture lasting approximately 110 minutes, followed by 10 minutes of communications between the teacher and students. After class, the teacher uploaded the PowerPoint slides used for the lecture to the e-learning platform. Outside of class, the students were free to self-study based on the corresponding textbook chapters.

### Course evaluation methods

Seven days after the teaching unit was conducted using the different teaching methods in the two groups, the students were synchronously given a test; this test aimed to assess their knowledge and level of understanding regarding the topic. In the test, the students in the two groups received the same questions but in a different order. The primary aim of the test was to assess the ability of the students to analyze typical questions on pharmaceutical marketing concerning the core knowledge that was prepared in advance by the teacher. The students’ knowledge and ability were measured by 5 short open-ended questions, 10 multiple-choice questions and 2 essay questions. The essay questions consisted of a short pharmaceutical marketing case. The students had to conduct a comprehensive analysis on this case. The short open-ended questions included the following: (1) What is the role of pharmaceutical representatives in pharmaceutical marketing activities? (2) How can a medical product be positioned in the minds of consumers, and what attributes can differentiate it from competitor products? (3) What are the two main purposes of market segmentation? (4) How are the commonly used criteria for market segmentation selected? (5) How are key opinion leaders identified through direct inquiry of physicians?

The total time for the test was fifty-five minutes. Students took three minutes and fifteen minutes to answer each multiple-choice question and essay question, respectively. Previously, we invited two educational experts to assess the two tests to ensure the integrity of the content and consistency in difficulty levels. The test scores were statistically analyzed to compare the differences between the two groups in knowledge and ability.

### Primary outcomes

#### The outcomes of teacher-student interaction

To evaluate the impact of the FC on the interactive activities between the teacher and students inside the classroom, we adopted Flanders’ interaction analysis system (FIAS), which was developed by Flanders (1962) to analyze teacher-student verbal behaviors in the context of classroom communication. The FIAS is a standardized observation method of interaction analysis that describes teacher-student interaction through predefined activity categories. In the FIAS system, ten categories of verbal statements were defined, with 7 categories for teacher talk, 2 categories for student talk and 1 category for silence or chaos in the FC and LBL classroom [45]. The FIAS has been employed by education researchers worldwide as an effective measuring tool for analyzing teacher-student [15, 45–48].

As shown in Table 1, we made a minor modification to the FIAS by adding “answering students’ questions” as a teacher talk category. Traditionally, Chinese college students have been regarded as passive learners, and they rarely ask questions in class [12]. To discover the impact of the FC on students’ in-class activities, we added some categories to analyze the percentage of student-initiated talk in class. Thus, “student-initiated talk: initiating a conversation between groups” (9b), “student-initiated talk: answering other group members’ questions” (9c) and “student-initiated talk: discussing problems within groups” (9d) were added as student talk categories. According to the following scheme, two trained RAs recorded the frequency of each category code of verbal behaviors in the class every 3 seconds. Further data were processed into so-called activity indices, which express teacher-student interaction as follows:

1. The percentage of teacher talk: This activity index was calculated by the ratio of the frequencies of the teacher’s verbal behaviors (category codes 1–7) to the frequencies of the total verbal behaviors (category codes 1–10) inside the classroom.
2. The percentage of student talk: This activity index was calculated by the ratio of the frequencies of the students’ verbal behaviors (category codes 8–9) to the frequencies of the total verbal behaviors (category codes 1–10) inside the classroom.
3. The percentage of silence or chaos: This activity index was calculated by the ratio of the frequencies of silence or chaos (category code 10) to the frequencies of the total verbal behaviors (category codes 1–10) inside the classroom.
4. The ratio of the teacher’s indirect influence to the teacher’s direct influence (I/D ratio): This activity index was calculated by the ratio of the frequencies of the teacher’s indirect influence (category codes 1–4) to the frequencies of the teacher’s direct influence (category codes 5–7) inside the classroom.
5. The ratio of the teacher’s positive influence to the teacher’s negative influence (P/N ratio): This activity index was calculated by the ratio of the frequencies of the teacher’s positive influence (category codes 1–3) to the frequencies of the teacher’s negative influence (category codes 6–7) inside the classroom.
6. The ratio of the students’ positive responses to the students’ passive responses: This activity index was calculated by the ratio of the frequencies of students’ positive responses (category codes 9a-9d) to the frequencies of students’ negative responses (category code 8) inside the classroom.

**Table 1:** The modified teacher-student verbal behavior coding scheme.

Category		Category code	Content	Detailed description
Teacher talk	Indirect influence	1	Accepting students’ feeling in a constructive way	① Accepting and clarifying students’ feelings (positive and negative) ② Expecting and reflecting on students’ feelings
		2	Giving encouragement or praise	① Verbal behaviors ② Nonverbal behaviors (nodding or signaling student to go on)
		3	Valuing student views	① Clarifying students’ views ② Commenting on student ideas
		4	Posing questions	① Posing questions to students ② Expecting students’ answers
	Direct influence	5a	Giving lectures	① Giving lectures ② Elaborating the teacher’s opinions ③ Quoting other authorities’ ideas
		5b	Answering students’ questions	
		6	Giving instructions	Giving directions, orders or commands
		7	Criticizing students or establishing authority	① Optimizing students’ behaviors using statements ② Criticizing students ③ Explaining the teacher’s behaviors (verbal and nonverbal)
Student talk		8	Teacher-initiated talk	① Responding to the teacher ② Responding to student peers
		9a	Student-initiated talk: giving lectures on a topic	① Expressing students’ opinions ② Initiating a new topic
		9b	Student-initiated talk: initiating a conversation between groups	
		9c	Student-initiated talk: answering other group members’ questions	
		9d	Student-initiated talk: discussing problems within groups	
		10	Silence or confusion	① Unclear communication ② Unable to observe the communication

We analyzed a total of 240 teaching minutes (120 minutes in the FC group and 120 minutes in the LBL group) using the modified method FIAS, and the results were graphically and statistically processed.

### Secondary outcomes

Regarding their attitudes, learning experiences and satisfaction under each teaching method, the students in both the FC and LBL groups were measured with the same self-administered questionnaire, which used a 5-point Likert scale.

A focus group interview that included teachers, students, teaching administrators and education scholars was conducted to collect the student attitude, learning experience and satisfaction attributes. In addition, some questions were adopted from previously published studies, such as those by Zeng *et al*. [33], Meng *et al*. [18] and Lin *et al*. [19].

The questionnaire contained 20 items and used a 5-point Likert scale, and the levels of agreement or satisfaction ranged from “strongly disagree” and “very dissatisfied” (“1”) to “strongly agree” and “very satisfied” (“5”). To collect the students’ total learning time investment outside of class, we designed a fill-in-the-blank questionnaire.

To evaluate the impact of the FC model on student learning gains in a more robust manner, we employed a linear regression model and controlled for student demographic variables, especially GPA [49]. The model for the regression is as follows:

$$Y_{final}=\beta_{0}+\beta_{1}\times Y_{PA}+\beta_{2}\times GPA+\beta_{3}\times Performance+\beta_{4}\times Intervention+\varepsilon$$

We define the following variables for each student: Y _final_ is the student’s score on the test; Y_PA_ is the student’s score on the prior pharmacy management course, which is the proxy for the pretest on pharmaceutical marketing; GPA is the student’s GPA; Performance is the student’s grade in the prior introductory course in pharmacy; and Intervention is a variable for whether the student was in the FC group (Intervention = 1 if the student was in the FC group; otherwise, Intervention = 0). In this study, each control variable (PA, GPA and Performance) was mean-centered by subtracting the mean of each variable from each student’s value [49].

### Statistical analysis

All statistical analyses were performed using the SPSS 17.0 statistical software package (SPSS, Inc., Chicago, IL, USA). Continuous variables were reported in terms of their mean and corresponding standard deviation (SD) or standard error (SE), while categorical data were presented as percentages.

The scores of the test results and the learning time outside of class invested by the students were used for an independent-samples t-test to analyze between-group differences. Due to their distribution characteristics, the differences between the two groups were compared through appropriate nonparametric tests. When appropriate, Mann-Whitney U tests were used to compare questionnaire values between the FC and LBL groups. The relationships between variables were examined using linear regression analysis. Multiple linear regression analysis was used to test the proposed model. The independent variables were the influences on final scores in the proposed model, and they were entered simultaneously. A t-test of the beta coefficient was used to test each hypothesis. All tests were two-tailed and were assessed at the 5% level of significance.

### Ethical considerations

This study was approved by the Nanjing Medical University Institutional Review Board (NJMUIRB (2018) 008). Written informed consent was obtained from all students.

### Patient and public involvement

No patients were involved in this study. The study participants were offered feedback on the study results and will be informed of this publication.

## Results

### Baseline student characteristics

The demographic distribution of the participants is presented in Table 2. The baseline variable data for the FC and LBL groups were examined via chi-square and two-tailed t-tests. The results showed that there were no significant differences by gender (χ^2^ = 0.440, *p* = 0.507), age (t = -0.298, *p* = 0.766), and GPA (t = -1.447, *p* = 0.09).

**Table 2:** Demographic data of the students who participated in the study.

Groups	Age	Gender ratio (male: female)	Grade point averages
FC group (n = 81)	21.51±0.82	29:52	3.50
LBL group (n = 56)	21.46±0.79	17:39	3.38
t or χ^2^ value	-0.298	0.440	-1.447
*p* value	0.766	0.507	0.09

In addition, there were 517 pharmacy students in the School of Pharmacy at NMU; the proportion of enrolled students accounted for 26.5% of the total number of pharmacy students at NMU. There were no significant differences between the sample and the total population in any of the variables (*p*>0.05). Thus, the sample could be considered representative of the population in this study.

### The test scores between the two groups

As shown in Table 3, the total test scores of the students in the FC group were 88.21±5.95, which were significantly higher than those of the students in the LBL group (80.05±5.59). The differences in the scores of the short-answer questions and essay questions were also significant (*p* = 0.000). However, there was no significant difference between the two groups in the scores of the multiple-choice questions (*p* = 0.502).

**Table 3:** Test scores between the two groups.

	FC group (mean±SD)	LBL group (mean±SD)	t value	*p* value
Short-answer questions	23.38±1.87	20.59 ±2.81	-6.50	0.000
Multiple-choice tests	8.25±1.93	8.04±1.60	-0.674	0.502
Essay questions	56.58±4.52	51.43±4.24	-6.73	0.000
Total final scores	88.21±5.95	80.05±5.59	-8.08	0.000

As presented in Table 4, the multiple linear regression model showed an R^2^ value of 0.838, *p*<0.001. The students’ score on pharmacy management (Y_PA_), their score in the prior introductory course in pharmacy (Performance) and whether they were in the FC group (Intervention) all had a significant influence on the students’ total final scores. Put differently, the “intervention” is significantly correlated with student performance, controlling for the influence of student demographic variables.

**Table 4:** Coefficients for students’ total final scores using linear multiple regression.

Model variables	Coefficient estimate	SE	*p* value
Intercept (β_0_)	80.05	0.38	*<*0.0001***
Y_PA:_ student’s score on pharmacy administration (β1)	0.64	0.08	*<*0.0001***
GPA (β_2_)	-0.28	0.51	0.585
Performance: student’s score in the introductory course in pharmacy (β_3_)	0.29	0.08	0.001**
Intervention (β_4_)	8.16	0.50	*<*0.0001***

* Significant at *p*<0.05

** significant at *p* < 0.01

*** significant at *p* <0.001

n = 137, R^2^ = 0.838. The F value for the overall model is (F = 171.086, *p*<0.001)

The estimated intercept (β_0_ = 80.05) means that the expected score on the final test for a student with an average Y_PA_, average GPA, and average Performance and who did not receive the intervention is 80.05.

The coefficient on Y_PA_ (β_1_ = 0.64) means that, holding all other variables constant, a student’s score on the final test is expected to increase by 0.64 points for each additional point the student scored on the prior pharmacy administration course.

The coefficient on Performance (β_3_ = 0.29) means that, holding all other variables constant, a student’s score on the final test is expected to increase by 0.29 points for each additional point in the student’s grade on the prior introductory course in pharmacy.

The coefficient on Intervention (β_4_ = 8.16) means that, holding all other variables constant, the score on the final test of a student who was taught via the FC teaching method is expected to increase 8.16 points compared to a student who did not receive the FC intervention.

### Comparison of the teacher-student interaction of the two groups

To better understand the teacher-student interaction results, we performed a comparative analysis of teacher-student verbal behaviors between the two groups based on the modified FIAS.

**Fig 2:**
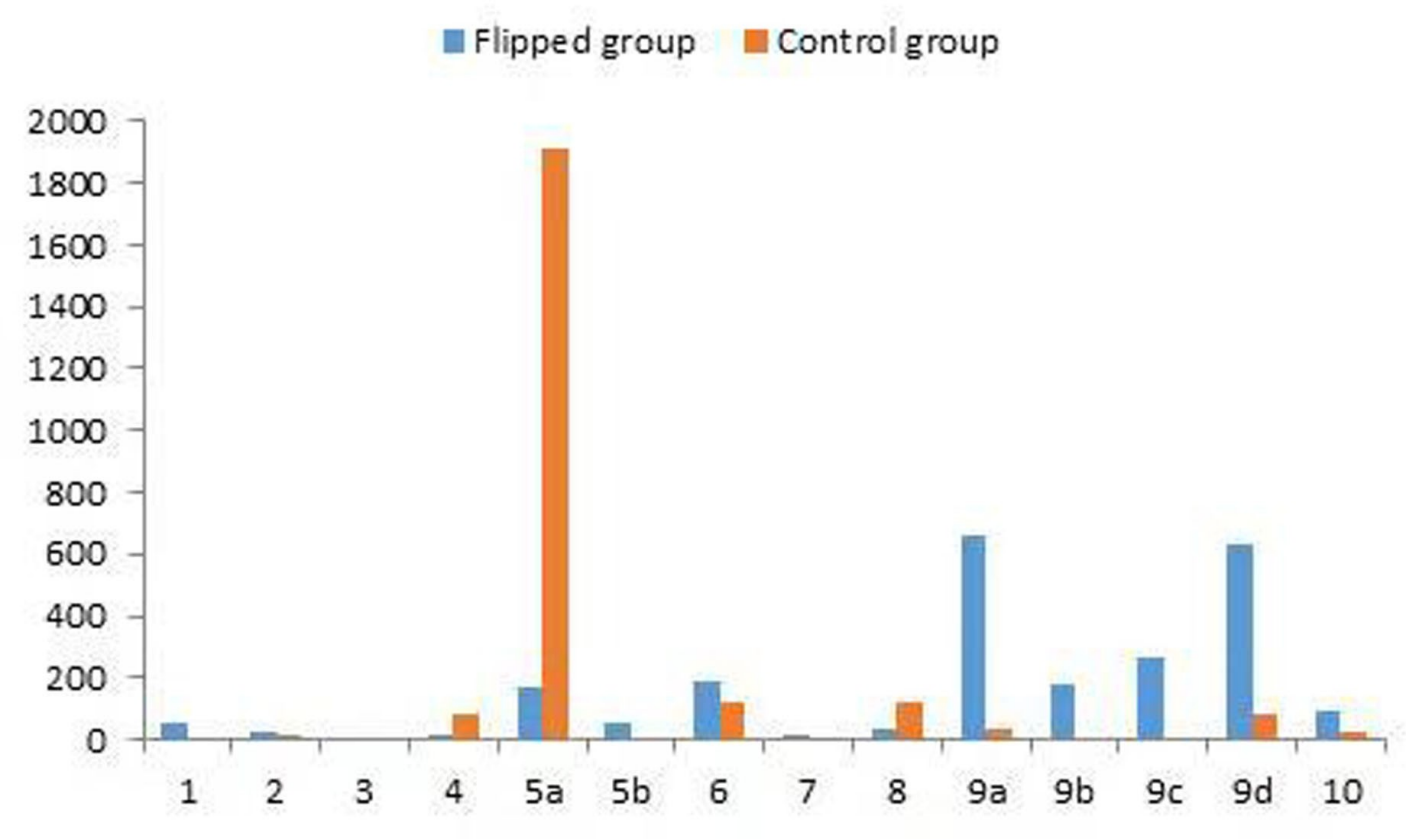
Verbal behavior frequencies in the intervention group and control group. Verbal behavior category codes: 1 accepting students’ feelings in a constructive way, 2 giving encouragement or praise, 3 valuing student views, 4 posing questions (teacher), 5a giving lectures, 5b answering students’ questions, 6 giving instructions, 7 criticizing students or establishing authority, 8 teacher-initiated talk, 9a student-initiated talk, 9b initiating a conversation between groups (students), 9c answering other group members’ questions (students), 9d discussing problems within groups,10 silence or chaos.

Structured observations of the verbal behaviors in the two groups were graphically recorded (Fig 2). In general, the number of observations of the teacher giving lectures (category code = 5a) in the control group was 1916, which dominated most of the verbal behaviors in the LBL class. In addition, six category codes of verbal behaviors were not recorded in the control group. Fig 2 shows in the LBL class, lecturing was the dominant activity of the teacher.

On the other hand, we compared the number of observations of student talk. The total number of observations of students giving lectures on a topic during the presentations (category code = 9a) in the FC group was 663. The other seven groups could interrupt the lecture and initiate a conversation between groups (category code = 9b), and the number of observations was 177.

According to the questions to be initiated by the other groups, the number of observations of answering other group members’ questions (category code = 9c) was 268. The total number of observations for student-initiated talk consisting of lecturing other students during the presentations was 445 (category codes = 9b, 9c). In addition, all the students could discuss problems within groups in FC, especially for solving the problems and exercises (Category code = 9d), the number of observations for group-based discussion was 635. All these numbers of observations accounted for a large percentage of the verbal behaviors in the FC. Thus, the results show that the students played the most active role in verbal communications with the students and teacher. Table 5 displays the frequencies and activity indices of the teacher-student verbal behaviors in the two groups. The results of the chi-square test of independence show that the percentage of teacher talk in the FC and LBL groups were 21% and 96%, respectively, presenting a significant difference (*p* = 0.000). However, the percentages of student talk in the FC and LBL groups were 75% and 2.6%, respectively, also exhibiting a significant difference (*p* = 0.000). This result indicates that student talk far exceeded teacher talk in the FC group. Notably, the number of observations for other types of teacher talk, such as category codes 1, 2, 3, 4 and 7, was relatively low in the two groups. The percentages of silence or chaos in the FC and LBL groups are also shown in Table 5; they are both very low. There were detectable differences between the two groups (4% vs. 1%, χ^2^ = 41.880, *p* = 0.000).

**Table 5:** The activity indices of the teacher-student verbal behaviors in the two groups based on the modified FIAS.

Activity indices	FC group (% or ratio)	LBL group (% or ratio)	Chi-square values	*p* values
Teacher talk	21%	96%	2170.274	.000
Student talk	75%	2.6%	2012.483	.000
Silence or chaos	4%	1%	41.880	.000
The ratio of the teacher’s indirect influence to the teacher’s direct influence	104:426 (1:4.10)	88:2046 (1:23.25)	152.496	.000
The ratio of the teacher’s positive influence to the teacher’s negative influence	88:205 (1:2.33)	3:120 (1:40)	38.602	.000
The ratio of the students’ positive responses to the students’ passive responses	1743:32 (54.47:1)	115:126 (0.91:1)	748.567	.000

In relation to the teacher’s verbal behavior, the I/D ratios in the FC and LBL groups were 1:4.10 and 1:23.25, respectively, showing a significant difference (*p* = 0.000). Then, we analyzed the students’ verbal behavior; compared with the LBL group, the FC group had a higher ratio of the students’ positive responses to the students’ passive responses (54.47:1 vs. 0.91:1, *p* = 0.000).

### Student attitudes toward and satisfaction with the teaching model

The overall evaluation results of the questionnaires are shown in Table 6 and Table 7. As shown in Table 6, the students in the FC group showed higher mean scores on the 8 attitude attributes (*p* = 0.000).

**Table 6:** FC vs. LBL group: Student attitudes toward the teaching model.

No.	Items	FC group (mean±SE)	LBL group (mean±SE)	*p* value
1	The teaching model of this course stimulates my positivity and initiative	4.00±.081	3.30±.067	.000
2	The teaching model of this course helps me analyze and solve problems.	3.94±.077	3.86±.065	.366
3	The teaching model of this course promotes my thinking and practice ability.	4.15±.083	3.12±.063	.000
4	I accept the teaching method of this course.	4.28±.071	4.20±.100	.607
5	The teaching model of this course improves my self-learning ability.	4.22±.105	3.30±.072	.000
6	I completely understand and master the knowledge of this course.	3.44±.129	3.39±.170	.979
7	The teacher gives opportunities for each student to express his or her views equally.	4.28±.083	3.50±.067	.000
8	When I have a different opinion from that of my teacher, the teacher provides effective guidance.	4.26±.078	3.34±.166	.000
9	When I cannot answer questions in class, the teacher encourages me.	4.36±.090	4.14±.082	.111
10	The questions that were raised by the teacher in the class are open, which can lead to discussion.	4.42±.068	3.09±.053	.000
11	My classmates usually stated their opinions in the class.	4.02±.080	2.59±.150	.000
12	The teacher encourages and praises the students.	4.28±.077	3.09±.069	.000

Scores are based on a 5-point Likert scale (1 = strongly disagree to 5 = strongly agree).

**Table 7:** FC vs. LBL group on satisfaction with the teaching model.

No.	Items	Flipped group (mean±SE)	LBL group (mean±SE)	*p* value
1	The interactions between the teacher and students	4.36±.069	3.05±.145	.000
2	My performance in this class	3.65±.100	3.59±.139	.961
3	My attitudes toward learning in this course	3.83±.099	2.88±.096	.000
4	The teacher’s attitude toward teaching in this course	4.54±.063	4.36±.093	.131
5	The teacher’s preparatory work in this course	4.48±.068	3.20±.123	.000
6	The teaching objective of this course	4.36±.077	3.41.124±	.000
7	The teacher’s performance in this course	4.43±.068	4.41±.087	.982
8	The effectiveness of the teaching method for this course	4.41±.070	3.29±.083	.000

Scores are based on a 5-point Likert scale (1 = very dissatisfied to 5 = very satisfied).

Compared with the students in the LBL group, the overwhelming majority of the students in the FC group held more positive attitudes toward their teaching model.

Table 7 summarizes the results of comparing the median scores of the 8 satisfaction attributes between the FC and LBL groups with Mann-Whitney U tests. Comparing the two groups, we found that there were significant differences in the rating of satisfaction with teacher-student interaction (*p* = 0.000), the students’ learning attitude (*p* = 0.000), the teacher’s preparatory work (*p* = 0.000), the teaching objective (*p* = 0.000), and the teaching effect (*p* = 0.000). Furthermore, for the abovementioned five satisfaction attributes, the FC group participants had significantly higher ratings than the LBL group participants.

### The time investment in learning between the two groups

**Fig 3:**
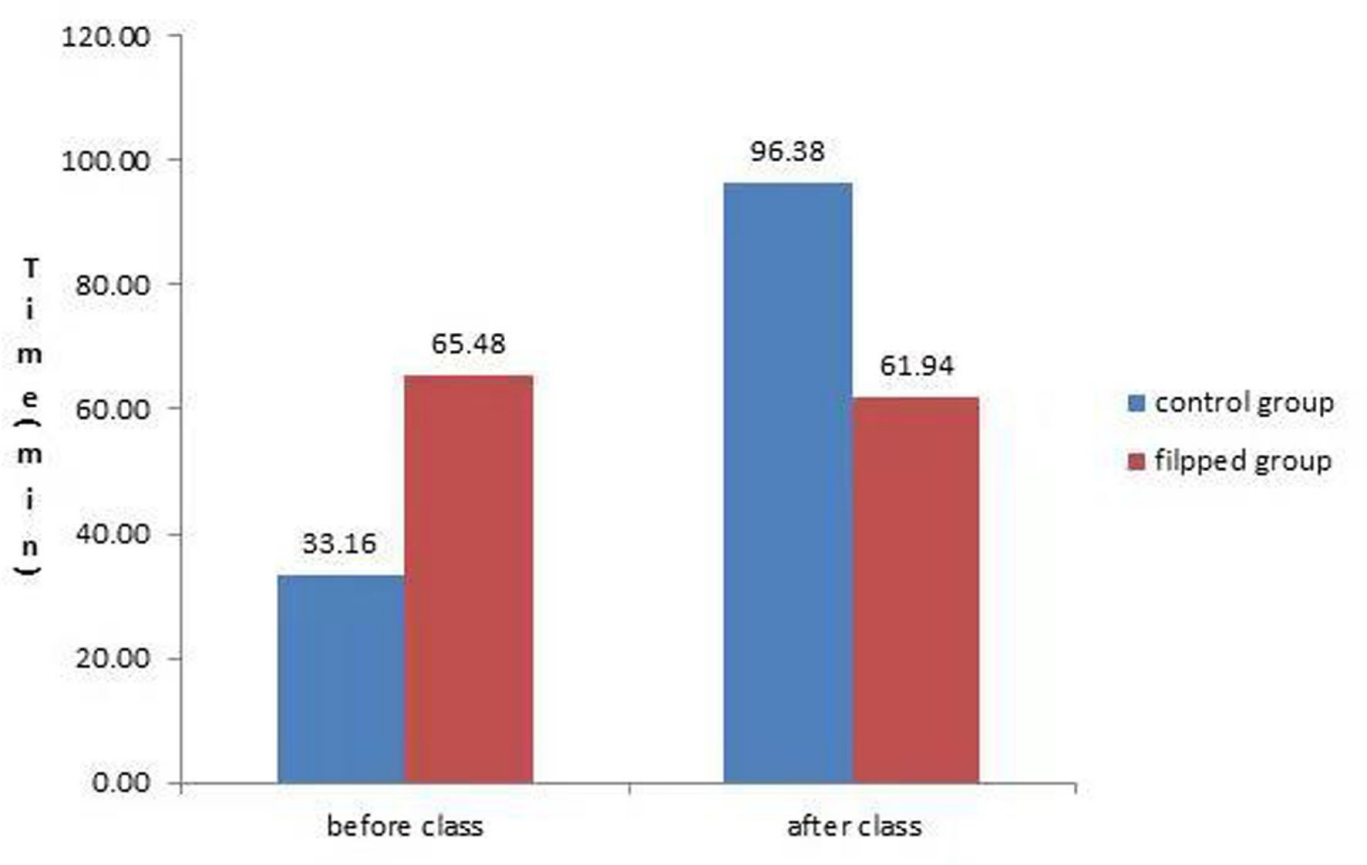
Differences in learning time after class between the FC Group and LBL group.

As shown in Fig 3, before the class, the students in the FC group invested significantly more time in their studies than those in the LBL group (65.48 ± 11.89 vs. 33.16 ±11.56, t = -15.90, *p* = 0.000). However, by contrast, after class, the students in the LBL group devoted significantly more time than did those in the FC group (96.38 ± 16.96 vs. 61.94 ± 10.04, t = 14.90, *p* = 0.000).

## Discussion

This study designs and provides a reference for flipped learning to improve teacher-student interaction and students’ learning outcomes. The primary goal of this study was to evaluate the effectiveness of the FC pedagogical model for a pharmaceutical marketing module. Specifically, this exploratory clustered randomized controlled study focused on the status of teacher-student interaction in the FC context, resulting in the following important findings.

In this study, traditional teacher-dominated lectures, during which students traditionally take notes, were replaced by an FC teaching model with highly interactive teacher-student activities. Prerecorded microvideo lectures, the main objective of which was the delivery of knowledge, were viewed independently by students prior to class. The scheduled lecture time was primarily devoted to consolidating student knowledge and developing student skills through in-class activities. Active student-centered learning, through student group presentations and group discussions, increased the opportunities for students to apply their knowledge and skill to pharmaceutical marketing case scenarios in class.

### The impacts of the flipped classroom model on teacher-student interaction

Some scholars and educators have argued that frequent and meaningful teacher-student interaction in the classroom within a higher education context could play a critical role in students’ learning and effectively assist their career development [50]. Moreover, a student-centered model underscored by active learning would necessitate class participation and interaction. In recent years, there has been increasing evidence of the promotion roles played by teacher-student interaction in the Chinese cultural context [51–52]. The findings of this study, shown in Table 5, confirm that the teacher held strict control over the verbal behaviors in the LBL classroom. In contrast, some results show that in the FC, student activity prevails due to the large percentage of student talk in the FC group. As shown in Table 5, from the results of the ratios of the students’ positive responses to the students’ passive responses between the two groups, the percentage of student-initiated talk in the FC group far exceeds that in the LBL group. We conclude that the students in the FC group had sufficient opportunities to express their opinions. In the FC group, students expressed their opinions through presentations, initiated conversations between groups and actively directed discussion sections on new topics. In addition, the students spent significant time answering other group members’ questions and discussing issues within groups, which could imply that there was a relatively enthusiastic atmosphere for communication activities among the students. The findings from this educational study could challenge previous conclusions, which claimed that most Chinese college students only took notes, listened carefully and seldom attempted to impose their ideas on other students in class [53–54].

Furthermore, the I/D ratio is very high (1:23.25) in the LBL class, which is another indication of the teacher’s initiative in controlling the activities in the LBL class. Meanwhile, in the FC group, the I/D ratio could be reduced to a lower level (1:4.10). Although this finding with regard to the I/D ratio is still not ideal, we can demonstrate that there was a relatively democratic relationship between the teacher and students in the FC group. These findings can provide a possible way to change the classroom atmosphere in Chinese cultural settings.

Interestingly, the teacher never or seldom showed verbal behaviors of criticizing students or establishing authority (category code = 7) in the two groups, demonstrating that the teacher never criticized students or tried to change students’ activities during the teaching process.

### The impacts of the flipped classroom teaching model on student academic performance

The findings from this study also support other educational research that reports improved academic performance associated with FCs [26–28,33]. Notably, the linear regression results provide robust evidence supporting the hypothesis that the FC model has a significant impact on student performance, controlling for the influence of other student demographic variables. In other words, even when controlling for a student’s score on the precourse, GPA and prior grades on performance, the FC intervention is still a predictive factor of the student’s score on the final test.

More specifically, based on the results of the students’ scores, we conclude that the FC model is more effective for achieving academic performance than is the LBL model. The reasons are as follows: In the test in this course, the purpose of the multiple-choice questions was to assess the knowledge points that the students acquired. Simultaneously, we established short-answer questions and essay questions to measure both the students’ knowledge and their application skills. On the one hand, the students in the FC group were better at the short-answer questions and essay questions than their counterparts in the LBL group. On the other hand, there were no significant differences in the scores of the multiple-choice questions between the two groups. This result is extremely important, as it means that the increase in scores was just an increase in performance on the final test items related to the topics of the presentations and discussions in the FC group. In the FC group, each group presented on a particular topic in class. Most of the short-answer questions and essay questions were tightly associated with the issues that the students discussed before or during the class, such as the criteria for market segmentation. As a result, the students in the FC group might have had better insights into the open-ended questions in the exam, which helped them prepare for and answer the questions on the final test. Due to the more desirable learning effects of the FC method, the FC gave students ample opportunities to acquire knowledge and apply pharmaceutical marketing skills. This result is in line with other studies [26, 55–59]. The rationale behind the FC teaching model is to promote effective teacher-student interaction and to stimulate “exploratory, group discussion-based and interactive” teaching activities in the classroom [60]. Chinese teachers should build a good environment for undergraduate students to communicate with one another and establish a positive cooperative relationship among group members to obtain a desirable learning effect.

### The impacts of the flipped classroom teaching model on students’ time investment

Compared with the LBL model, the students exposed to the FC model invested more time and energy before the class. Conversely, the students exposed to the LBL method invested more time after class than those in the FC group. The possible reasons are as follows. The students in the FC group usually invested plenty of time and energy in searching for information in network databases, watching microvideos and discussing issues with peers before class. The increased investment in self-learning prior to class could be a key factor in the higher level of understanding achieved by the students in the FC group [56, 59]. Furthermore, they could also benefit from the Q&A sessions with the teacher and from class discussions with peers in the FC. Therefore, due to their familiarity with the teaching contents, the students in the FC group did not need to spend much time and effort on knowledge review after class. However, the students in the LBL group just reviewed the textbook, course materials, PowerPoint files and class notes for self-learning outside of class. Under the traditional LBL model, the lack of enough opportunities for the students to apply their knowledge and skills in the classroom may have been one of the reasons that a serious amount of time was invested in review after class.

### The impacts of the flipped classroom teaching model on student attitudes and satisfaction

The findings of the questionnaire survey revealed that the students in the experimental group expressed more positive attitudes toward the FC model. The following could be a possible explanation for this finding. First, as one of the most helpful learning tools, the FC method enabled the students’ individualized learning before and after class. The process of delivering knowledge was set before class in the FC environment; therefore, the students had an opportunity to select their study patterns based on their learning habits. For example, according to their time plan, they could easily follow the online course material, such as the teaching microvideos, anytime and anywhere. Meanwhile, prerecorded lectures could be assigned to the students as homework, leaving classroom time open for interactive learning activities. Subsequent to the students’ independent acquisition of content, engaging in knowledge integration through teacher-student interaction and collaboration with other students is a hallmark of the FC. Conversely, in the LBL environment, the teacher considers the learning status of only the majority of the class, and as a result, the personalized learning needs of the students are neglected. Because different students have different study habits and abilities, student-centered teaching models, such as the FC model, can boost each student’s cognitive abilities by allowing the student to decide his or her learning schedules according to his or her own specific needs. This is a key contributing factor for students to achieve better learning outcomes [56].

Second, the proportion of cognitive learning in the FC group, such as understanding and memorizing outside class, was lower than that in the LBL group. Conversely, the vast majority of learning activities under the FC model were carried out through cooperative learning or problem-based learning; thus, the teaching activities were accomplished through teacher-student interaction and cooperation between peers [26, 57, 61]. The students preferred interactive class time more than in-person lectures. Most of the time in the FC was invested in developing high-level cognitive learning abilities that could stimulate the students’ intellectual abilities to analyze, think and solve problems. Compared with the LBL teaching method, the FC is better able to stimulate the learning interests of students, increase their self-control abilities, and more effectively guide their self-learning outside of class [26, 55,27]. Similarly, the findings of this study agree with those of previous studies [56, 59, 27]. In summary, the satisfaction of individual learning needs, the decrease in cognitive learning activities, and the improvement in one’s intellectual abilities and learning interest are possible reasons why students generally have a higher degree of satisfaction with FCs.

### Strengths of this study

The current study is the first to use the FC pedagogical model for the STP strategy chapter of pharmaceutical marketing in China. The use of a previously validated FIAS methodology allows the measurement of teacher-student interaction in a reliable and standardized manner. Furthermore, this FC project discovered that the quantity of teacher-student interaction is a compelling force in improving student performance and satisfaction with the teaching model. Thus, this study can serve as reference for further innovative teaching models and to promote the application of FCs.

### Limitations of this study

However, this study has some limitations. First, in this study, only one test was used to examine student performance, which is insufficient to validate the chronicity of the acquisition of knowledge and skills. Therefore, the results should be generalized with caution. Future research should employ controlled studies that objectively repeat the test to identify the possible long-term effects throughout a semester. Second, this study tested only a fraction of course content, limiting the generalizability of the results to the entire pharmaceutical marketing course system. Future studies on the FC are needed to verify the validity of the results claimed in this study by testing large-scale and more in-depth teaching contents. Third, the positive results obtained cannot be attributed solely to the FC pedagogical model. Therefore, a larger sample size and a more rigorous design should be employed to evaluate these results. Fourth, the Hawthorne effect and sample selection bias may also be potential factors that influence student learning [62], which may threaten the reliability of these results. Thus, the results should be generalized with caution. Educators who may apply the FC pedagogical model should be aware of the contextual factors and individual differences regarding experiences and achievements.

## Conclusion

The current study provided an innovated concept and framework in pharmacy education in the Chinese cultural context. Compared with LBL methods, the implementation of the FC pedagogical model in this study improved student performance, increased teacher-student interaction and generated positive student attitudes toward the experience. As an effective teaching model, it can also stimulate pharmacy students’ learning interest and enhance their self-learning abilities. Further research is necessary to validate the efficacy of the FC model.

## Supporting information

S1 File Students baseline data. (XLS)

S2 File GPA data. (XLS)

S3 File Test scores data. (XLS)

S4 File Out-of-class study time data. (XLS)

S5 File Teacher-student interaction data in FC group. (XLS)

S6 File Teacher-student interaction data in LBL group. (XLS)

S7 File Questionnaire data on students attitudes. (XLS)

S8 File Questionnaire data on students satisfaction. (XLS)
